# Adherence to the Mediterranean Diet in Maltese Adults

**DOI:** 10.3390/ijerph18010010

**Published:** 2020-12-22

**Authors:** Sarah Cuschieri, Massimo Libra

**Affiliations:** 1 Department Anatomy, Faculty of Medicine and Surgery, University of Malta, MSD2090 Msida, Malta; 2 Department of Biomedical and Biotechnological Sciences, University of Catania, 95123 Catania, Italy; m.libra@unict.it; 3 Research Center for Prevention, Diagnosis and Treatment of Cancer, University of Catania, 95123 Catania, Italy

**Keywords:** mediterranean diet, traditional dietary pattern, western diet, nutrition transition, adherence, Malta

## Abstract

Background: Populations living in Mediterranean islands are experiencing a nutrition transition process from traditional to Westernized dietary patterns. No information on this matter regarding individuals living in Malta have been published to date. The aim of this study was to assess the level of adherence of the Maltese people to the Mediterranean diet and which factors were associated with it. Methods: A nationwide cross-sectional study was conducted in the island of Malta between 2014 and 2016. A literature-based Mediterranean diet adherence score was used to assess the level of adherence to the dietary pattern. Results: Out of 3947 adults, the overall Mediterranean diet adherence score mean was 7.19 (SD 1.91): being female, non-smoker, and having older age was associated with higher adherence to the Mediterranean diet. Less clear pattern of association was found for educational and occupational status, for which medium educational level and a high occupational level were associated with lower adherence to Mediterranean diet. Higher adherence was finally associated with consumption of healthier food groups (more rice and dark bread and less pasta and white bread; more all plant-food groups and fish, less animal-food sources, including fast foods; more light cheeses and yogurt were more frequently consumed among higher adherent individuals in spite of regular ones). Conclusions: Adherence to the Mediterranean diet in Malta is lower than in those of populations living in companion Mediterranean islands. Given the lack of data on this topic, further studies should be conducted among the Maltese people and public health nutrition interventions should be planned to improve current eating habits toward more traditional dietary patterns.

## 1. Introduction

The Mediterranean diet has been the focus of research for many scientists over the last decades due to its potential for preventing several non-communicable diseases [1]. This dietary pattern finds its origin in the traditional dietary habits handed down over the millennia by individuals living in the Mediterranean Basin, an area characterized by a fervent trading of olives and wine, great variety of fruits and vegetal species imported from other geographical regions of the world and easily acclimatized in the area [2]. However, it was not before the 1960s that the nomenclature “Mediterranean diet” was coined by Ancel Keys while studying the risk factors for coronary heart disease of individuals living in southern Italy. In that occasion, the peculiar lifestyle habits of such a population, in contrast with that of the United States, seemed, and later confirmed, to decrease the risk of coronary heart disease, corroborating the idea that such lifestyle would prevent chronic diseases [3]. The dietary pattern characterizing the habits of southern Italian populations had some specific features that could be summarized as follows: (i) high intake in plant-based foods, such as fruit and vegetables, but also whole-grain cereals and legumes, nuts and seeds, olive oil as main dressing and added fat, and moderate consumption of (red) wine consumed during meals; (ii) moderate intake of animal products, such as fish and poultry, milk and dairy foods, and eggs; and (iii) low consumption of red (processed) meat [4]. The following fifty years have been characterized by a deepening of research in this area, showing that a diet with such features would be associated with reduced risk of cardiovascular disease [5,6], metabolic disorders [7,8], neurodegenerative diseases [9], and certain cancers [10]. Eventually, the Mediterranean diet has been declared “Intangible Cultural Heritage of Urgent Safeguarding” by the United Nations Educational, Scientific and Cultural Organization (UNESCO), while the scientific community agreed that dietary patterns inspired by the Mediterranean diet may be beneficial for human health also in a non-Mediterranean context [11]. Finally, the Mediterranean dietary pattern has been also proven to be a sustainable choice for the environment [12,13,14].

While most scientific evidence and available reports on Mediterranean diet adherence refer to Italian, Greek, and Spanish islands when considering the Mediterranean Basin [15], adherence rates have been reported to be consistently lower in most populations worldwide [16,17], including those in the Mediterranean basin [18,19]. The Maltese Islands are found at crossroads between Sicily and Libya with an archipelago covering a total surface area of 316 km^2^, making it one of the smallest European nations with a total population of 493,559. Over the years, a number of studies have reported the high increase in the occurrence of obesity, diabetes, and other cardiometabolic diseases among the adult population in Malta [20,21,22,23]. While nutritional risk factors have been universally considered as major determinants of non-communicable diseases [24,25], only a few studies published dietary data on Maltese population [26,27,28]. Compared to other Mediterranean islands, Malta has preserved its original culture less intensively while better adapting to other cultural influences over the centuries [27]. The last foreign occupation by the British has had a tremendous impact on several aspects of the society, including Maltese food system and dietary habits [29]. As a result, a previous study investigating the level of adherence to the Mediterranean diet among Mediterranean islands revealed that Malta had the lowest adherence to this dietary pattern along with the lowest consumption of fish and fruit [28]. However, no other study has been published so far on this issue and current dietary habits of the Maltese population are largely unknown, while results from national surveys suggest a growing rise in metabolic disorders [30]. Thus, the aim of this study was to investigate the level of adherence to the Mediterranean diet and associated determinants of people living in Malta.

## 2. Materials and Methods

### 2.1. Study Population

A nationwide cross-sectional study was conducted in Malta island between 2014 and 2016 by following a health examination survey protocol. The detailed protocol can be found elsewhere [31]. Briefly, a randomized single stage sampling technique was followed to establish the sample population from a national register. A single stage sampling technique could be followed due to the small total population size (<500,000) and small geographical location (316 km^2^). The population sample was stratified by age (18 to 70 years) and gender living in each town in order for the total population to represent approximately 1% of each residential town. All individuals living in Malta for at least 6 months and holding a permanent identification permit were eligible. However, those individuals who were pregnant, too ill to attend the health examination hubs or living abroad at the time of the study were excluded. Invitations to the randomised population were sent out by post and those accepting to participate in the study were asked to visit their local peripheral health clinic fasted for at least 9 hours on a set day and time. Out of 3947 individuals invited, data from a total of 1861 participants was finally collected. A weighting factor was applied to the study population in order to maintain representation by considering each gender and age per residing town. The adjusted study population (*n* = 3947) was used for descriptive statistics while the unadjusted study population (*n* = 1861) was used for analytic statistics. 

The Research Ethics Committee of the Faculty of Medicine and Surgery at the University of Malta (Ethics code 19/2014) together with the Information and Data protection commissioner gave their permission for this study. All participants gave their informed written consent to participate in the study.

### 2.2. Data Collection

As part of the health examination survey, each participant was invited to take part in an interviewer-led validated questionnaire [31]. The interviewers were physicians and trainee medical doctors trained throughout the study period. The questionnaire gathered socio-economic data (demography, education, occupation), lifestyle characteristics (smoking, alcohol intake, physical activity), and a food frequency questionnaire. The participants’ ages were subdivided into four age groups representing typical family generations. Beverage (such as tea, coffee, sugar-sweetened) and supplements data were not gathered. The educational status was categorised into (i) low (≤10 years), (ii) medium (11–13 years), and (iii) high (≥14 years). The occupation status was categorised into (i) unemployed, (ii) low (unskilled professions), (iii) medium (skilled professions), and (iv) high (managerial professions). Smoking status was defined as no smoking, current smoking, and former smoking, while physical activity categorisation was adopted from Merwether et al. assessment tool and grouped as (i) sedentary (less than 30 min of activity per week), (ii) low (walk at least for 30 min per week), (iii) moderate (30–60 min of physical activity other than walking per week), and (iv) high (>60 min of physical activity other than walking per week) [32].

### 2.3. Mediterranean Diet Adherence Assessment

Dietary information was included in the questionnaire used and frequency consumption of 29 food/beverage groups was investigated. The food frequency for each food item was converted into servings per day. The MEDI-LITE Mediterranean Diet adherence score was calculated through a literature-based score where consumption of fruit, vegetables, legumes, cereals, fish, meat and meat products (beef/veal, pork, poultry, fish, eggs, sausages and burgers were considered in this study), dairy products, alcohol, and olive oil portions per day were used to estimate the level of adherence. The study population Mediterranean diet score was then divided into tertiles and labeled as (i) low (≤6 score), (ii) medium (7–8 scores), and (iii) high (≥9 score). Of note, as part of the dairy products, the consumption of the Maltese traditional cheese known as “Gbejniet” (cheeselets, generally produced by pasteurized goat’s milk) was collected as a separate food item.

### 2.4. Statistical Analysis

Continuous variables were presented as means and standard deviations; Student’s t-test and one-way ANOVA for independent samples were used. Categorical variables were analysed using Chi squared test. In order to evaluate the association between demographic, socio-economic and lifestyle characteristics and adherence to the Mediterranean dietary pattern, regression analysis was performed by considering the Mediterranean diet score as the dependent variables (high vs. medium/low adherence). The results were presented as odds ratios with the corresponding 95% confidence interval. A *p*-value of <0.05 was considered as statistically significant. All analyses were conducted using IBM SPSS^®^ version 21 software (IBM Corp. Released 2012. IBM SPSS Statistics for Windows, Version 21.0. Armonk, NY, USA).

## 3. Results

The total adjusted study population was 3947 adults (18 to 70 years) with a slightly higher proportion of male (50.6%) and a mean age of 44.8 (SD 15.1) years. The overall Mediterranean diet adherence score mean was 7.19 (SD 1.91). The distribution of the study population demographic, socioeconomic, and lifestyle factors by tertiles of adherence score to the Mediterranean diet is shown in Table 1. A higher proportion of women scored in the highest tertile compared to men; similarly, individuals in the older age groups scored higher adherence to the Mediterranean diet (Table 1). Differences by educational and occupational status were also found, albeit with no clear trends: individuals in the highest and lowest educational categories had a higher proportion of high adherence to the Mediterranean diet; moreover, those in the highest category of occupational status had lowest adherence (Table 1). A higher mean adherence to the Mediterranean diet was also observed among those with healthier lifestyle habits including no smoking and increased physical activity (Table 1).

A number of demographic, socioeconomic, and lifestyle habits exhibited an association with adherence to the Mediterranean diet, as shown in Table 2. Being female was associated with 69% odds of higher adherence to Mediterranean diet than their counterparts. Similarly, increase in age exhibited a higher association with adherence to the Mediterranean diet. A medium educational level and a high occupational level were both associated with lower adherence to Mediterranean diet; similarly, low physical activity and smoking habits (current and former) were associated with lower adherence.

The adherence to the Mediterranean diet was further evaluated in accordance to the different food groups as shown in Table 3: individuals reporting higher adherence to the Mediterranean diet consumed more rice and dark bread and less pasta and white bread; all plant-food groups (fruit and vegetables) were more consumed in the high-adherent group while all animal-food sources, except fish, and fast foods were less consumed; among dairy products, light cheeses and yogurt were more frequently consumed among higher adherent individuals in spite of regular ones (Table 3).

## 4. Discussion

The present study aimed to investigate the dietary habits of Maltese population and to assess the level and the socio-demographic determinants of adherence to the Mediterranean diet. Compared to previous studies using this score [18,33,34], the adherence rate to the Mediterranean diet can be considered relatively low. However, the adherence was associated with some socio-demographic variables investigated.

Demographically, older women, non-smoker, unemployed, were more adherent to the Mediterranean diet. This result is generally in line with previous studies conducted in Mediterranean islands [33,35,36,37] and for the first time reported concerning the Maltese population. Younger generations have been often reported to be less likely to follow a Mediterranean dietary pattern [38,39,40,41,42,43]. The globalization process and the so-called “Westernization” of the diets have led to a slow, yet growing abandonment of traditional dietary habits in the Mediterranean countries together with the introduction of high-calorie, nutrient-poor ultra-processed foods [44]. This phenomenon, identified as a “nutrition transition” from traditional to globalized dietary patterns, is accompanied by other issues, such as scarce availability of local and seasonal foods and limited economical accessibility due to elevated costs for the recommended foods [45]. While in non-Mediterranean countries a higher adherence to this dietary pattern may be the result of a choice reflecting a health-conscious profile, we hypothesize that in Mediterranean islands, this adherence is only the result of a higher adherence to traditional lifestyle behaviours and maintenance of the cultural heritage. This hypothesis has been reported in other studies conducted in Mediterranean islands, where identification of higher adherence among unemployed women may depend on the social role of older generations of mothers in the management of the families, including preparation of meals [46,47,48]. In contrast, other studies yet conducted in Mediterranean countries reported that higher adherence to this dietary pattern was associated with a higher economic allowance, suggesting that the economic crisis occurred in the last decade and the rise in cost of healthier foods may have played a role in the observed abandonment of this traditional dietary pattern [49,50]. Both interpretations may not necessarily be in contrast: in fact, in our study, adherence to the Mediterranean diet has been related to the occupation of individuals; it is noteworthy to underline that occupation does not necessarily imply the economic status of a person, rather can be considered as a proxy variable. The economic crisis that affected Italy over the last years may have modified the salary of individuals: a number of conditions may affect the real economic disposability of individuals irrespectively of their occupational class (i.e., non-skilled workers having highly profitable commercial activity versus highly skilled workers underpaid by the system under crisis). As a result, evaluation of the occupational status may not reflect the real income of participants.

The Mediterranean dietary pattern has evolved over time, changing across countries and adapting to the available foods. This dietary pattern does not stand for a unique list of ingredients and foods, but rather is differently represented across Mediterranean countries although maintaining the common features previously listed. Concerning Malta, individuals more adherent to the Mediterranean had an obvious higher consumption of food groups characterizing the dietary score used (i.e., fruits, vegetables, legumes, fish) and lower of those considered not related to it (i.e., meat products); interestingly, regarding food groups unrelated to this dietary pattern, individuals reporting higher adherence had also less consumption of refined bread, ready-made meals, pizza/burgers, and full milk, accompanied with higher intake of brown bread, skimmed milk and light yogurt, suggesting food preferences toward a healthier diet. However, compared to previous reports on Maltese dietary habits conducted in the past, we recorded some differences. An early study published about 15 years ago conducted on two generations of women (mothers and daughters) showed an increase in legumes, meat products, poultry, and vegetables compared to the past [27]. In contrast, another study published later on contrasted with our findings, showing higher weekly intake of fruit, vegetables, and legumes, and lower intake of meat products (despite the study not distinguishing between poultry and red meat) [28]. Based on the timing of data collection, we may consider our findings as current dietary habits, especially relative to the newest generations of Maltese people. These findings may be the result of an overall larger availability of food variety and higher access to a greater diversity of foods by the whole population, leading to higher consumption of nutrient-rich foods, such as meat products, but also lower consumption of plant-derived foods. This hypothesis reflects the finding related to the low levels of adherence to the Mediterranean diet observed among individuals in the highest occupational category compared to the unemployed. Considering that Maltese dietary habits have never been displayed as fully meeting the Mediterranean diet because of the Anglo-Saxon influences, our findings depict an evolving scenario in which Maltese people are moving forward a more Westernized diet as well as the rest of Mediterranean islands while losing the dietary improvements observed in the last few generations.

The results of this study should be considered in light of some limitations. The information retrieved may suffer from recall bias and declared data may not correctly reflect real intakes. Moreover, the questionnaire used was not able to capture detailed information regarding other aspects of the Mediterranean diet often underrated and unexplored, such as the use of herbs, spices, and flavors that are known to contribute to the health benefits of this dietary pattern.

## 5. Conclusions

In conclusion, while scientific evidence and cultural recognition have emphasized the role of the Mediterranean diet for humans, current evidence suggests that modern generations are shifting away from this dietary pattern in favor of more Westernized diets. Malta is no exception to such trends, reporting an adherence rate to the Mediterranean diet lower than those of populations living in companion Mediterranean islands. Given the lack of data on this topic, further studies and investigations should be conducted among the Maltese people and public health nutrition interventions should be planned to improve current eating habits toward more traditional dietary patterns.

## Figures and Tables

**Table 1:** Mediterranean diet adherence scores by demographic, socioeconomic, and lifestyle characteristics of the study sample (*n* = 3947).

Variables	Mediterranean Diet Score, Mean (SD)	Mediterranean Diet Score Tertiles, *n* (%)			*p*-Value
		T1—Low (≤6) (*n* = 1498)	T2—Medium (7–8) (*n* = 1489)	T3—High (9+) (*n* = 960)	
**Gender**					<0.001
Male	7.45 (1.96)	871 (58.2)	736 (49.5)	391 (40.8)	
Female	6.93 (1.81)	627 (41.8)	753 (50.5)	569 (59.2)	
**Age group, *n* (%)**					<0.001
<30	7.11 (1.83)	327 (21.9)	283 (19.0)	201 (20.9)	
30–44	6.89 (1.87)	507 (33.8)	422 (28.4)	209 (21.7)	
44–65	7.36 (1.92)	536 (35.7)	646 (43.3)	431 (44.9)	
>65	7.47 (1.97)	128 (8.6)	138 (9.3)	120 (12.5)	
**Educational level, *n* (%)**					<0.001
Low	7.38 (1.88)	220 (14.7)	232 (15.5)	183 (19.1)	
Medium	6.73 (1.94)	944 (63.0)	882 (59.3)	498 (51.9)	
High	7.16 (1.81)	334 (22.3)	375 (25.2)	279 (29.0)	
**Occupational level, *n* (%)**					0.002
Unemployed	7.25 (2.99)	149 (10.0)	201 (13.5)	120 (12.5)	
Low	7.25 (1.77)	120 (8.0)	111 (7.5)	90 (9.3)	
Medium	7.27 (1.88)	386 (25.8)	432 (29.0)	272 (28.3)	
High	7.27 (1.88)	842 (56.2)	744 (50.0)	479 (49.9)	
**Smoking status, *n* (%)**					<0.001
Non-smoker	7.40 (1.83)	765 (51.1)	858 (57.6)	615 (64)	
Current smoker	7.03 (1.94)	460 (30.7)	316 (21.2)	183 (19.1)	
Former smoker	7.40 (1.83)	273 (18.2)	315 (21.2)	162 (16.9)	
**Physical activity level, *n* (%)**					0.004
No activity	7.39 (1.91)	134 (9.1)	145 (9.8)	108 (11.2)	
Low	7.02 (1.91)	309 (20.6)	267 (17.9)	176 (18.3)	
Medium	7.15 (1.86)	905 (60.4)	921 (61.9)	542 (56.4)	
High	7.43 (2.08)	148 (9.9)	155 (10.4)	135 (14.1)	

**Table 2:** Association between demographic characteristics and high adherence to Mediterranean diet (high vs. medium/low).

Variables-	High Adherence OR (95% CI)
**Gender**	
Male	1
Female	1.69 (1.51–1.90)
**Age group**	
<30	1
30–44	0.79 (0.67–0.93)
44–65	1.25 (1.07–1.46)
>65	1.40 (1.12–1.74)
**Educational level**	
Low	1
Medium	0.74 (0.63–0.87)
High	1.02 (0.85–1.22)
**Occupational level**	
Unemployed	1
Low	0.94 (0.72–1.22)
Medium	0.91 (0.75–1.11)
High	0.77 (0.64–0.92)
**Smoking status**	
Non-smoker	1
Current smoker	0.57 (0.49–0.65)
Former smoker	0.82 (0.70–0.95)
**Physical activity level**	
No activity	1
Low	0.79 (0.63–0.99)
Medium	0.86 (0.70–1.04)
High	1.17 (0.91–1.50)

**Table 3:** Consumption (servings per day) of selected food groups by level of adherence to the Mediterranean diet.

Food Item	Mediterranean Diet			*p*-Value
	Total	Medium/Low	High	
**Carbohydrates, mean servings/d (SD)**				
Potatoes	0.31 (0.22)	0.32 (0.21)	0.31 (0.22)	0.26
Pasta	0.25 (0.16)	0.25 (0.16)	0.23 (0.16)	<0.001
Rice	0.14 (0.15)	0.13 (0.15)	0.16 (0.16)	<0.001
White Bread	0.46 (0.36)	0.48 (0.35)	0.40 (0.35)	<0.001
Brown Bread	0.14 (0.26)	0.12 (0.25)	0.18 (0.28)	<0.001
**Plant food sources, mean servings/d (SD)**				
Salad / raw vegetables	0.30 (0.27)	0.27 (0.26)	0.40 (0.30)	<0.001
Boiled vegetables	0.29 (0.25)	0.25 (0.22)	0.44 (0.28)	<0.001
Vegetables in hot dishes	0.18 (0.21)	0.15 (0.19)	0.28 (0.26)	<0.001
Legumes	0.02 (0.08)	0.01 (0.06)	0.03 (0.12)	<0.001
Fruits	1.97 (1.47)	1.71 (1.41)	2.81 (1.33)	<0.001
Vegetable or Vegetarian dishes	0.21 (0.25)	0.18 (0.24)	0.30 (0.29)	<0.001
**Animal food sources, mean servings/d (SD)**				
Beef/Veal	0.16 (0.14)	0.16 (0.14)	0.15 (0.13)	0.001
Pork	0.12 (0.12)	0.13 (0.12)	0.12 (0.12)	0.033
Poultry	0.29 (0.19)	0.29 (0.19)	0.30 (0.20)	0.171
Fish	0.14 (0.15)	0.12 (0.14)	0.22 (0.15)	<0.001
Egg	0.16 (0.18)	0.16 (0.18)	0.15 (0.17)	0.007
Ready-made meals	0.09 (0.15)	0.10 (0.16)	0.06 (0.12)	<0.001
Pizza/Burgers	0.11 (0.13)	0.12 (0.14)	0.10 (0.11)	<0.001
Sausages	0.07 (0.12)	0.07 (0.12)	0.05 (0.10)	<0.001
**Dairy foods, mean servings/d (SD)**				
Light Cheese	0.10 (0.21)	0.09 (0.21)	0.11 (0.21)	0.064
Cheese	0.32 (0.30)	0.33 (0.30)	0.28 (0.30)	<0.0001
Cheeselet (“Gbejniet”)	0.14 (0.18)	0.13 (0.17)	0.15 (0.19)	0.005
Ricotta	0.10 (0.15)	0.10 (0.15)	0.11 (0.15)	0.232
Skimmed Milk	0.41 (0.41)	0.39 (0.41)	0.46 (0.41)	<0.001
Full Milk	0.26 (0.38)	0.29 (0.38)	0.20 (0.35)	<0.001
Light Yogurt	0.16 (0.28)	0.14 (0.27)	0.23 (0.32)	<0.001
Yogurt	0.06 (0.17)	0.06 (0.18)	0.05 (0.16)	0.096
Mozzarella	0.07 (0.12)	0.07 (0.12)	0.07 (0.12)	0.521
Alcohol (standard drink)	0.29 (0.19)	0.29 (0.19)	0.30 (0.20)	0.181

## Data Availability

The data presented in this study are available on request from the corresponding author. The data are not publicly available due to ethics and data protection re-strictions.
